# Hydrogel-Forming Microneedles in the Management of Dermal Disorders Through a Non-Invasive Process: A Review

**DOI:** 10.3390/gels10110719

**Published:** 2024-11-07

**Authors:** Popat Mohite, Abhijeet Puri, Shubham Munde, Nitin Ade, Ashwini Kumar, Pensak Jantrawut, Sudarshan Singh, Chuda Chittasupho

**Affiliations:** 1 AETs St. John Institute of Pharmacy and Research, Palghar 401404, Maharashtra, India; mohitepb@gmail.com (P.M.); abhijeetp@sjipr.edu.in (A.P.); shubhamvmunde@gmail.com (S.M.); nitinade2@gmail.com (N.A.); 2 Research and Development Cell, School of Engineering and Technology, Manav Rachna International Institute of Research and Studies, Faridabad 121003, Haryana, India; drashwinikumar.research@gmail.com; 3 Faculty of Pharmacy, Chiang Mai University, Chiang Mai 50200, Thailand; pensak.j@cmu.ac.th; 4 Office of Research Administration, Chiang Mai University, Chiang Mai 50200, Thailand

**Keywords:** biocompatible, biodegradable, dermal disease, hydrogel, microneedles, non-invasive

## Abstract

Microneedle (MN) technology has emerged as a promising approach for delivering therapeutic agents to the skin, offering significant potential in treating various dermal conditions. Among these technologies, hydrogel-forming microneedles (HFMNs) represent a transformative advancement in the management of dermal diseases through non-invasive drug delivery. These innovative devices consist of micrometer-sized needles made of native or crosslinked hydrophilic polymers, capable of penetrating the stratum corneum without damaging underlying tissues. Upon insertion, HFMNs rapidly absorb interstitial fluid, swelling to form a hydrogel conduit that enables the efficient transport of therapeutic agents directly into the dermal microcirculation. The non-invasive nature of HFMNs enhances patient compliance by eliminating the pain and discomfort associated with traditional hypodermic needles. This technology allows for the delivery of a wide range of drugs, including macromolecules and biomacromolecules, which are often difficult to administer dermally due to their size and polarity. Moreover, HFMNs provide controlled and regulated release profiles, enabling sustained therapeutic effects while minimizing systemic side effects. Additionally, HFMNs can be used for both drug delivery and real-time interstitial fluid monitoring, offering valuable insights into disease states and treatment responses. This dual functionality positions HFMNs as a versatile dermatology tool capable of effectively addressing various dermal complications. This review explores the potential use of polymeric biomaterials in HFMN fabrication and their application in treating major dermal disorders, such as acne, psoriasis, and other skin conditions. Furthermore, the review highlights the non-invasive nature of MN-based treatments, underscoring their potential to reduce patient discomfort and improve treatment adherence, as supported by the recent literature.

## 1. Introduction

The skin is the body’s largest and most versatile organ, providing essential protection, regulating temperature, and enabling sensory perception. It prevents water loss and blocks the entry of toxic compounds, allergens, irritants, and microbes [1]. Most dermatological disorders affect the skin’s first viable tissue layer, which can be impacted by various diseases and conditions, each with distinct causes and treatment approaches. The causes of skin disorders may include genetic factors, immune system disorders, illnesses affecting the thyroid or kidneys, diabetes, and infections from viruses, fungi, or bacteria trapped in hair follicles and skin pores. Other sources include a weakened immune system, contact with irritants or allergenic chemicals, exposure to infected individuals, and microorganisms, parasites, or fungi living on the skin [1]. A common dermal disorder is acne, which is caused by the blockage of hair follicles and is typically managed with topical treatments or medications [2]. Treatments for eczema, also known as atopic dermatitis, include topical steroids and moisturizers. This condition is characterized by itchy, inflamed areas of skin, often triggered by environmental factors and genetic predisposition [3]. Another common dermal condition is psoriasis, an autoimmune disease that leads to scaly patches and accelerated skin cell turnover [4,5]. It can be managed with topical medications, systemic treatments, or phototherapy [6]. Topical drug delivery effectively addresses superficial infections and disorders. However, for deep dermal therapeutic activity, drug permeation is a critical factor that must be considered. Therefore, developing an effective and safe delivery system is essential for treating a variety of skin conditions [7].

Liposomes and nanoparticles, developed using polymeric matrices, have shown significant efficacy in overcoming the skin barrier and facilitating drug absorption through the skin while allowing for controlled and prolonged medication release [8,9,10]. While these formulations address the challenges of targeted drug delivery systems, they have drawbacks, including a limited understanding of penetration mechanisms, scalability issues, and difficulty in regulating deeper drug penetration [11]. Novel methods have been reported to enhance drug permeability through the skin layers. Various physical approaches include hypodermic needles, iontophoresis, electrophoresis, and ablation. However, each method comes with its own advantages and disadvantages [12,13]. These methods may lead to skin allergies and irritation, require qualified personnel, and involve cost considerations. Additionally, they can compromise the skin’s natural barrier function over an extended period [14,15]. Microneedle (MN) technology presents a promising drug delivery method that can penetrate the skin with minimal discomfort [12]. Microneedles can create transient microchannels, allowing for the delivery of therapies that are unsuitable for transdermal or intradermal administration due to their large macromolecular size. Additionally, these small arrays of needles can also be used to diagnose infectious diseases [13]. Microneedles are self-managing devices capable of identifying biomarkers from the interstitial fluid of the skin in a minimally invasive manner, facilitating illness detection. These tiny, one-millimeter-diameter structures can access the dermal layers without touching blood vessels or pain-sensing neurons, offering painless, non-invasive, and controlled drug delivery for treating dermal disorders and infections. Microneedles effectively transfer molecules across the skin barrier, making hypodermic needle injections less uncomfortable. Furthermore, this non-invasive device reduces the risk of spreading blood-borne infectious diseases and minimizes damage from needle sticks [16]. Microneedles can be fabricated from various materials and are classified into several types: solid microneedles (MNs), coated microneedles, hollow microneedles, dissolving microneedles, and hydrogel-forming microneedles (HFMNs) [17].

Hydrogel-forming microneedles, an intriguing category of microneedles first reported in 2012, consist of swellable polymers, specifically crosslinked hydrogels such as chitosan, hyaluronic acid, sodium alginate, and poly(vinyl alcohol). These materials enable the sustained delivery of drugs over extended periods by either incorporating the drug into the polymer structure during preparation or by loading the drug into a separate reservoir and attaching it to the HFMNs [18,19,20,21]. Compared to conventional microneedles (MNs), hydrogel-forming microneedles (HFMNs) offer several advantages and represent a significant advancement in the field of dermal drug administration [22]. These cutting-edge tools combine the benefits of hydrogels with advanced technology, enhancing performance and broadening applications, particularly in the management of skin disorders. Conventional microneedles, made from silicon, polymers, or metals, primarily function by creating microconduits in the skin to facilitate the transport of medicinal substances. While traditional microneedles are effective, they have drawbacks, including the potential for irritation or skin damage due to their rigidity [23]. Typically, conventional microneedles rely on diffuse release or passive diffusion to administer medications, which can be ineffective and occasionally result in suboptimal therapeutic outcomes. In contrast, hydrogel-forming microneedles represent a paradigm shift due to their unique features and functionalities. Generally composed of hydrophilic and biocompatible polymers that swell in the presence of moisture, such as skin interstitial fluid, hydrogel microneedles are increasingly utilized in medical applications [24]. Thus, due to their ability to swell, hydrogel microneedles can form a gel-like matrix that penetrates the skin and serves as a reservoir for regulated drug delivery [25]. Comparing this method to conventional microneedles reveals several advantages, including enhanced comfort and reduced invasiveness. Additionally, hydrogel microneedles are softer and more flexible than standard materials, offering greater adaptability and comfort at the administration site [26]. In addition to treating skin conditions, hydrogel-forming microneedles (HFMNs) have been investigated for their potential to deliver biopharmaceuticals, gene therapies, and vaccines [27,28]. Hydrogel-forming microneedles offer several advantages over injections and invasive procedures. They are easy to administer, minimizing discomfort and anxiety associated with injections, particularly for patients who fear needles or require frequent dosing. Moreover, the familiarity and simplicity of using a pill or applying a patch can enhance patient acceptance of non-invasive methods, as individuals are generally more inclined to embrace these approaches.

Hydrogel-forming microneedles offer several benefits. The hydrogel matrix enables controlled and sustained release, providing a non-invasive alternative to injections. HFMNs have successfully delivered various drugs and therapeutic agents, including insulin, for managing diabetes [29]. Metformin has been delivered using hydrogel-forming microneedles (HFMNs) to provide sustained release, thereby enhancing patient compliance compared to traditional oral administration [29]. Moreover, hydrogel-forming microneedles (HFMNs) have been utilized to deliver vaccines, including those for influenza and hepatitis B, by targeting immune cells in the skin, which enhances immune responses [30]. Additionally, hydrogel-forming microneedles (HFMNs) have been used for the localized delivery of doxorubicin, a chemotherapeutic agent, directly to skin tumors, improving treatment efficacy while minimizing systemic side effects [31]. HFMNs have been used to deliver corticosteroids, such as dexamethasone, for treating inflammatory skin conditions like eczema and psoriasis, providing targeted delivery while reducing systemic effects [32]. However, several issues still need to be addressed, including potential biosafety concerns associated with long-term use, challenges in industrial production, and sterilization. The development of hydrogel materials that are both cost-effective and durable is essential for their widespread application. Additionally, further research is required to ensure the stability of the medication within the hydrogel matrix and to achieve reliable and consistent drug release [33]. Future developments are likely to focus on refining hydrogel compositions, exploring new production methods, and enhancing the overall functionality and applicability of hydrogel-forming microneedles (HFMNs). This review aims to provide a comprehensive analysis of HFMNs and their applications in managing skin disorders, addressing existing knowledge gaps. Additionally, it summarizes the fundamental principles underlying HFMNs, including structural and material composition, as well as an assessment of evaluation studies that confirm the integrity of HFMNs’ therapeutic applications.

## 2. Dermal Drug Delivery: A Versatile Approach for Skin-Targeted Therapies

**Figure 1:**
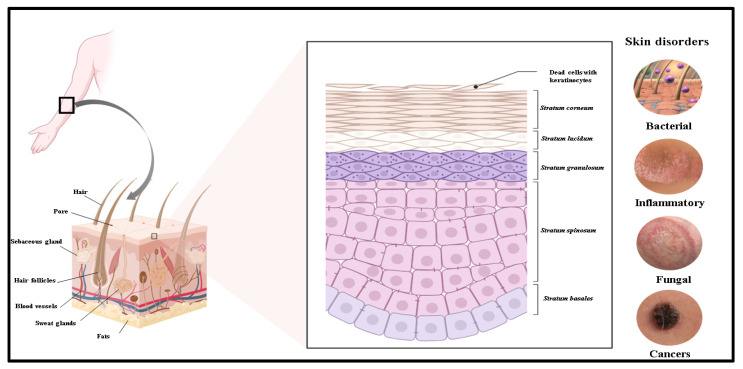
Illustration for anatomy of skin and related disorders. (The figure was created based on the authors’ own ideas using BioRender: ScientificImage and Illustration Software. Version 04).

Dermal drug delivery is a versatile method suitable for various formulations and applications. Its localized nature and reduced systemic side effects make it an attractive option for many medical conditions, especially those affecting the skin or requiring targeted treatment [34]. Human skin is the largest and most versatile organ in the body. It serves as a protective barrier, regulating temperature and sensory perception. The skin prevents water loss and protects against the potential penetration of toxic compounds, allergens, irritants, and microbes. Additionally, it functions as a complex physical barrier against ultraviolet radiation and particulate matter from the external environment [35]. The skin comprises three essential layers: the outermost epidermis, the dermis, and the subcutaneous tissue or hypodermis. The epidermis consists of five layers, with the topmost layer, the stratum corneum (SC), responsible for the skin’s barrier properties. The dermis contains connective tissues, blood vessels, lymphatic vessels, nerve endings, hair follicles, sebaceous glands, and eccrine glands. The subcutaneous tissue is composed of adipose cells and provides insulation to the body (Figure 1). The outer layer of the epidermis, known as the SC, acts as a barrier that restricts the penetration of molecules larger than 500 Da. The stratum corneum mainly consists of corneocytes surrounded by hydrophobic lipid layers that are approximately 10–20 μm thick. Beneath the SC lies the viable epidermis, composed of keratinocytes, melanocytes, Merkel cells, and Langerhans cells, with a thickness of 50–100 μm. Most dermatological disorders occur in this first viable tissue layer of the skin [36]. Skin-associated disorders, such as chronic wounds, psoriasis, eczema, acne, hyperpigmentation, skin aging, and vitiligo, affect millions of people worldwide [37]. Wound healing is often associated with common terms such as “scab”, which refers to a hard covering of dried blood that forms over a wound to protect the area as it heals, and “fibrotic skin”, a pathological condition characterized by excessive accumulation of fibrous connective tissue in the skin, primarily within the dermis. Additionally, “epithelial hypertrophy” describes the abnormal increase in the size or number of epithelial layers. This condition can occur in various tissues and is often a response to chronic irritation, inflammation, or injury.

Traditional dermal treatments typically involve topical or systemic therapies, which can be invasive or less effective. Non-invasive drug delivery systems, such as hydrogel-forming microneedles (MNs), offer promising alternatives by delivering targeted therapies directly to the affected skin layers. Chronic wounds and conditions such as diabetic ulcers, psoriasis, eczema, acne, hyperpigmentation, skin aging, and vitiligo can be effectively treated using MNs [38]. These microneedles (MNs) can deliver therapeutic agents such as growth factors, antimicrobial agents, and cytokines directly to the wound site, enhancing healing and reducing infection risks. Conditions like psoriasis, eczema, and atopic dermatitis can be effectively treated using hydrogel-forming microneedles (HFMNs) that deliver therapeutics such as corticosteroids or moisturizers directly into the affected skin layers, improving treatment efficacy while minimizing side effects. Additionally, hyperpigmentation disorders like melasma and age spots can be addressed with skin-brightening agents like vitamin C or kojic acid delivered directly to the epidermal–dermal layers, leading to improved pigmentation treatments and a more uniform skin tone. Furthermore, drug delivery for vaccines can benefit from HFMNs by stimulating robust immune responses while providing a more comfortable experience. In dermatological cancer treatments, HFMNs can deliver chemotherapeutic agents or immune modulators directly to cancerous lesions, enhancing treatment efficacy and minimizing systemic side effects.

## 3. Hydrogel-Forming Microneedles: A Promising Alternative for Controlled and Targeted Drug Delivery

**Figure 2:**
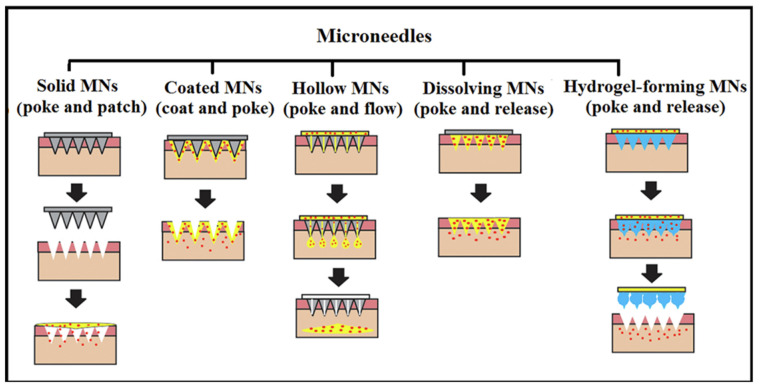
The drug delivery mechanism of solid microneedles (solid MNs), coated microneedles (coated MNs), hollow microneedles (hollow MNs), dissolving microneedles (dissolving MNs), and hydrogel-forming microneedles (hydrogel-forming MNs). Reproduced with permission from [44] under CC BY 4.0.

The unique properties of the skin make the microneedle (MN) delivery system a promising alternative to conventional drug delivery methods. Its defensive, inflammatory, and immunological characteristics contribute to this potential. Among various dermal drug delivery techniques, the MN-mediated system is defined as the non-invasive delivery of medications through the skin, and it has garnered significant attention from research institutions and companies alike [39]. These micron-scale needles have been shown to penetrate the skin through the stratum corneum (SC) into the viable epidermis while avoiding nerve fibers and blood vessels, which are primarily located in the dermal layer [40]. Moreover, these microneedles (MNs) are smaller and shorter than traditional hypodermic needles, making them less painful as they penetrate only the outer layer of the skin (the epidermis) and sometimes the upper layer of the dermis. This limited penetration activates fewer pain receptors, resulting in a sensation akin to a light prick or slight pressure. Additionally, MNs can be designed for quick insertion, minimizing discomfort duration and making the application less intrusive than that of a slower hypodermic needle. MNs are versatile, innovative devices available in various shapes, sizes, and materials, making them suitable for a wide range of applications. They can be designed to detect, diagnose, and treat numerous conditions by efficiently delivering vaccines, nanoparticles, proteins, antibodies, and drugs of varying molecular weights. These therapeutics or carriers can be precisely loaded into MNs to target specific skin layers, effectively reaching immune cells such as neutrophils, Langerhans cells, and dendritic cells, thereby promoting a robust immunological response [41]. Traditional microneedles (MNs) are categorized into four basic types based on their mechanisms: solid, coated, dissolving, and hollow (Figure 2). Each type offers specific benefits, such as controlled drug release or enhanced drug stability.

Hydrogel-forming microneedles represent an innovative advancement in microneedle (MN) technology, with the potential to address several limitations commonly associated with traditional MNs. These include significantly reduced drug-loading capacity, challenges in achieving precise drug coating, and difficulties in controlling the rate and extent of drug release [42]. These microneedles (MNs) are fabricated from biocompatible, water-responsive polymeric materials that form a gel upon insertion into the skin. Once inserted, hydrogel-forming microneedles (HFMNs) absorb surrounding fluids, causing them to swell and create microchannels in the skin. This process allows for controlled and sustained drug release while maintaining the needle’s structure, effectively providing a pathway for the medication to penetrate deeper into the dermal layers [43]. Their unique ability to hydrate and form a gel ensures efficient, regulated drug diffusion, particularly for hydrophilic compounds, without leaving any residual material in the skin after removal [33]. These controlled or regulated release mechanisms involve the absorption and swelling of drug molecules by body fluids, forming a gel-like layer that modulates drug diffusion. Some HFMNs also incorporate erosion-based mechanisms, wherein the matrix degrades over time, allowing for controlled drug release. Additionally, certain HFMNs can be designed to respond to specific environmental conditions, such as changes in pH or temperature, further regulating the release profile. The controlled release offers several therapeutic advantages, including improved bioavailability, reduced side effects, dosing flexibility, targeted delivery, and enhanced patient compliance. It can also decrease dosing frequency, particularly for chronic conditions, and simplify medication regimens through extended-release formulations. Overall, controlled release can improve the effectiveness of HFMNs in maintaining therapeutic levels and ensuring patient adherence. Moreover, HFMNs can deliver macromolecules with enhanced therapeutic efficacy when applied topically. The hydrogel matrix allows for sustained and controlled drug release, maintaining therapeutic levels over an extended period. This minimizes systemic side effects, which is especially beneficial for biomacromolecules that may cause adverse effects if absorbed into the bloodstream.

Hydrogel-forming microneedles (HFMNs) are integrated systems composed of two main components: an array of microneedles made from crosslinked polymers and a drug reservoir. This design enhances skin penetration, enabling deeper drug delivery into the dermis for localized treatment of various skin conditions. HFMNs are minimally invasive, resulting in less pain and discomfort compared to traditional delivery methods, which in turn improves patient compliance. The hydrogel matrix within HFMNs allows for controlled drug release, thereby enhancing therapeutic effects and prolonging the drug’s availability at the target site. By delivering drugs directly to the action site, HFMNs reduce systemic exposure, minimizing absorption and associated side effects, and thereby improving the overall safety profile of the treatment. Typically, the polymers used in HFMNs are hydrocolloids, such as cellulosic or cellulose derivatives, which can absorb significant amounts of interstitial fluid and swell upon insertion into the skin. Once the microneedles are inserted, they absorb interstitial fluid, creating a swollen polymeric matrix rich in capillaries. This process generates continuous microchannels that connect the drug reservoir to the surrounding tissue, facilitating enhanced drug delivery. The swollen matrix acts as a membrane that controls the drug release rate. After the drug delivery is complete, the empty microneedles can be removed from the skin without leaving any polymeric residue, providing greater flexibility in terms of shape and size. Additionally, the density of the hydrogel matrix can be adjusted to achieve specific drug release rates. However, despite their advantages, HFMNs may experience limitations, such as poor mechanical strength and physical stability, which could affect their performance and durability in practical applications [44].

**Figure 3:**
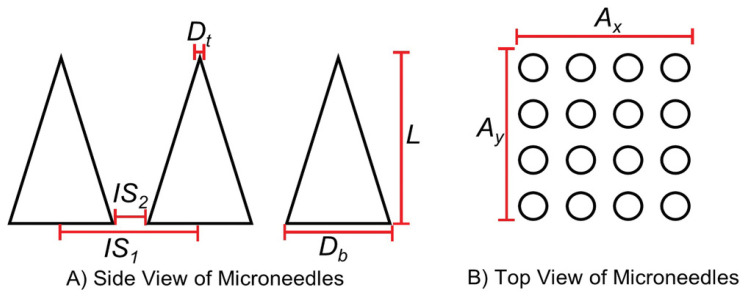
(**A**,**B**) Illustration showing the different dimensions taken into consideration for microneedle arrays. Reproduced with permission from [42] under CC BY 4.0.

Hydrogel-forming microneedles (HFMNs) significantly enhance patient compliance due to their reduced pain, ease of self-administration, and quick application. Their minimally invasive design makes them more comfortable compared to traditional injections, which encourages regular use among patients. HFMNs can be conveniently applied at home, removing the need for professional assistance, which is particularly beneficial for those with chronic conditions requiring frequent treatments. The fast application process typically takes only a few minutes, allowing patients to seamlessly integrate HFMNs into their daily routines with minimal disruption. The design features of HFMNs, including smaller tip diameters, lower tip angles, and higher tip height-to-width ratios, contribute to successful skin penetration. In studies comparing different base geometries of microneedles, it was found that HFMNs with triangular and square bases achieved greater penetration depths (340 μm and 343 μm, respectively) than those with hexagonal bases (197 μm). Specifically, the average penetration distance from the microneedle base plate to the stratum corneum was measured to be 660 μm for the triangle base, 657 μm for the square base, and 803 μm for the hexagonal base, indicating that the geometric design plays a crucial role in the effectiveness of drug delivery through the skin. This improved penetration capability further underscores the advantages of HFMNs in facilitating efficient and effective transdermal drug delivery [45]. A summary of design considerations is presented in Table 1 and Figure 3.

## 4. Fabrication Methods for Hydrogel-Forming Microneedle Arrays

### 4.1. Polymeric Materials Used in the Fabrication of Hydrogel-Forming Microneedles

**Figure 4:**
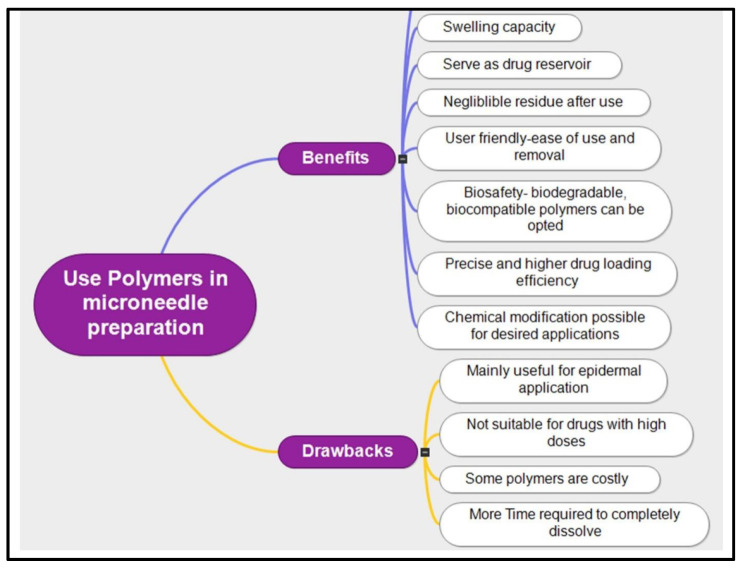
Benefits and drawbacks of polymeric MNs. Reproduced from [45] under CCBY 3.0.

Microneedles (MNs) function by penetrating the protective layers of human skin or mucosa, making it crucial that the materials used are biocompatible. Generally, hydrogels are biocompatible and can be used without causing harm or discomfort to the user. Several polymers employed in the fabrication of hydrogel-forming microneedles (HFMNs) have been extensively explored and tested for medical and dermatological applications, demonstrating essential properties such as biocompatibility and biodegradability. Figure 4 illustrates the benefits and drawbacks of various polymers used to fabricate MNs.

#### 4.1.1. Natural Polymers Fabricated Hydrogel-Forming Microneedles

Biocompatible polymeric materials derived from natural sources that can form hydrogels are commonly utilized in the fabrication of microneedles (MNs). In carbohydrate-based MNs, natural polysaccharides such as chitosan, cellulose, and starch are used to develop the hydrogel matrix. Protein-based MNs often incorporate gelatin or silk fibroin as the matrix-forming components. These MNs are characterized by their biocompatibility, biodegradability, and non-toxicity, exhibiting properties such as swelling capacity. Natural polymer-based hydrogel MNs have demonstrated promising results in drug delivery, wound healing, and dermal vaccination applications [46].

##### Chitosan and Chitosan Derivatives

Chitosan (CH) is an alkaline copolymer of glucosamine and N-acetylglucosamine derived from chitin, which originates from lower organisms such as certain algae, fungi, invertebrates, insects, and crustaceans [47]. This material is valued for its excellent biocompatibility, biodegradability, and antimicrobial properties, making it a key component in food and biomedical engineering. Chitosan derivatives are also extensively used in the fabrication of various drug delivery systems, cosmetics, and water treatment applications due to their enhanced water solubility and antibacterial activity, which result from the effective interaction of carboxymethyl groups with water and their natural biocompatibility [48].

Chitosan-based HFMNs have been investigated for transmucosal vaccine delivery, including through the mouth and nasal passages. In a study by Bobbala and Hook, an antigen was delivered using chitosan-based HFMNs to the oral mucosa in a rat model, resulting in a robust immune response. This finding suggests that HFMNs could be a viable method for oral vaccine delivery [49]. In Haojie Wei’s study, HFMNs were fabricated using chitosan and pullulan to enhance dermal drug delivery. The research involved two types of hydrogels: carboxymethyl chitosan–silk fibroin and oxidized pullulan, noted for their excellent swelling and water retention capabilities. These hydrogels were used to develop MNs loaded with *Salvia miltiorrhiza*, named HFMNs-1 (prepared using chitosan) and HFMNs-2 (prepared using pullulan). The results showed that HFMNs-1 had excellent mechanical properties and allowed for rapid drug release when applied to newborn porcine skin. These findings highlight the potential of natural polymer-based hydrogel MNs for effective, painless, and biocompatible dermal drug delivery [50].

##### Hyaluronic Acid

Hyaluronic acid (HA) is a glycosaminoglycan found in epithelial and dermal layers, as well as in connective tissue. Approximately 50% of the body’s total HA is located in the skin. The body degrades about one-third of its HA daily, replacing it with newly synthesized HA. Under normal physiological conditions, HA is converted to its more hydrophilic sodium salt form. Moreover, due to the numerous hydroxyl groups, HA can retain up to 1000 times its weight in water. This exceptional water-holding capacity supports various physiological functions and homeostatic processes in the body. HA-based microneedles (HA-MNs) are known for their high biocompatibility and resistance to deformation, and they are commonly used in skincare products [51].

Additionally, HA-based microneedles (HA-MNs) are robust enough to penetrate the skin effectively, dissolve quickly, and release drugs or active ingredients within a controlled, short time frame. The fabrication of HA-MNs does not require heating or organic solvents, which helps preserve and maintain the stability of heat-sensitive or chemically unstable agents, such as insulin [52]. Moreover, hyaluronic acid (HA) of varying molecular weights can impact the skin differently. High-molecular-weight HA has anti-aging effects, forms a protective film on the skin’s surface, and retains moisture, whereas medium-molecular-weight and low-molecular-weight HA can penetrate deeper into the skin, providing moisturizing benefits. Due to these advantageous properties, HA is a compelling choice as a base material for dermal delivery of active ingredients via microneedles (MNs) [53].

##### Sodium Alginate

Sodium alginate is a linear polysaccharide extracted from brown algae. It consists of 1,4-β-d-mannuronic (M) and α-L-guluronic (G) acids in varying proportions, resulting in a highly viscous aqueous solution. Due to its abundance of carboxyl and hydroxyl groups, sodium alginate exhibits significant chemical activity and can quickly form a hydrogel with a three-dimensional network structure. It is non-toxic, odorless, and known for its excellent biocompatibility and environmental friendliness. Sodium alginate has been safely used in dental applications, such as impression materials, since the 1950s. Additionally, sodium alginate and its derivatives are readily available, cost-effective, and renewable [54].

A study by Atefeh Malek-Khatab developed a new approach to treating acne using sodium alginate and gelatin microneedle (MN) patches loaded with clindamycin. The study focused on developing MNs with varying concentrations of clindamycin hydrochloride (6, 8, and 10 μmol/L) and assessed their mechanical strength, density, and drug release behavior. The results indicated that the addition of gelatin enhanced the mechanical properties and density of the MNs, while the concentration of clindamycin influenced the drug release profile. The MNs successfully penetrated pig cadaver skin and inhibited the growth of Cutibacterium acnes without harming normal human dermal fibroblast cells, suggesting a practical option for treating acne [53].

#### 4.1.2. Synthetic Polymers Fabricated Hydrogel-Forming Microneedles

Microneedles (MNs) fabricated using synthetic polymers offer advantages in controlled drug delivery due to their ability to dissolve or swell in response to moisture or heat. This responsiveness allows for the precise release of medications or other therapeutic agents into the body. Compared to natural polymer-based MNs, those made from synthetic polymers typically exhibit more consistent and robust mechanical properties, enhancing their overall effectiveness and reliability. Furthermore, synthetic polymers can be tailored to achieve specific characteristics, such as adjustable degradation rates, which adds flexibility to their applications in drug delivery systems.

##### Poly(vinyl) Alcohol

Poly(vinyl) alcohol is a water-soluble, polar synthetic polymer known for its excellent biocompatibility and biodegradability, along with its inherent non-toxicity and favorable mechanical properties. Poly(vinyl) alcohol can be crosslinked either chemically or physically to fabricate hydrogels. Additionally, due to their wound-healing capabilities, which involve the upregulation of transforming growth factor beta, poly(vinyl) alcohol hydrogels are frequently employed as matrix materials in wound dressings [55]. Samant and Prausnitz demonstrated the capability of crosslinked poly(vinyl) alcohol HFMNs to extract interstitial fluid. In their study, the HFMNs achieved an extraction rate of 0.0030 µL per MN volume after 12 h of use on porcine skin, compared to a 0.0039 µL extraction rate using micropores after just 20 min. These findings further validated that crosslinked poly(vinyl) alcohol, when used alone without additional polymers, exhibits a slower swelling rate [56]. In another study, Tang et al. modified a 10 wt. % poly(vinyl) alcohol formulation by incorporating Cy5.5 and poly(ethylene glycol) methyl ether amine. This modification enhanced both the water solubility and water retention of the HFMNs. In addition, the inclusion of Cy5.5, a commonly used dye, facilitated the fluorescence-based detection of the HFMNs during application [57].

##### Methacrylate-Based Hyaluronic Acid

Chemical modification of HA with methacrylic acid (MA) yields methacrylate-based hyaluronic acid (HAMA). This modification results in a composite-based hydrogel characterized by a continuous three-dimensional network structure, which imparts excellent swelling properties, mechanical strength, and drug-loading capacity. The HAMA hydrogel maintains high stability under simulated human physiological conditions (pH 7.4) [58]. Xu et al. utilized HAMA to develop an HFMN patch for managing diabetic ulcers. This HFMN patch, which incorporates platelet-derived growth factor D and human adipose-derived stem cells, was tested in a diabetic mouse model and demonstrated efficacy in promoting angiogenesis and enhancing wound healing [59].

##### Gantrez S-97

Grantrez S-97 is a versatile hydrophilic polymer primarily composed of methyl vinyl ether and maleic anhydride. Its structure includes vicinal dicarboxylic acid functionalities, which enhance its chelating properties for metal ions such as iron, magnesium, zinc, and calcium. Moreover, this polymer is recognized for its unique properties, making it suitable for various applications, particularly in the pharmaceuticals, personal care, and food industries. A study reported by the Donnelly group using Grantrez S-97, PEG, and sodium bicarbonate demonstrated robust and effective penetration with extensively swellable HFMNs [60].

### 4.2. Techniques Involved in the Fabrication of Hydrogel-Forming Microneedle Arrays

Hydrogel-forming microneedles can be easily fabricated using various methods, depending on the hydrogel-forming polymeric materials and the crosslinking mechanisms employed [61]. As outlined in the previous subsections, the materials include both natural and synthetic polymers. These polymers can undergo physical crosslinking, electrostatic interactions, or chemical crosslinking to form hydrogels, which are subsequently shaped into microneedle patches, as described in this section.

**Figure 5:**
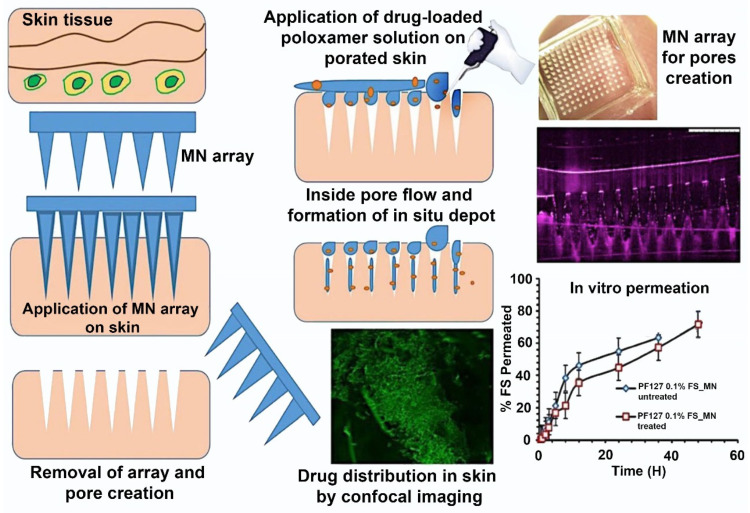
Illustration for development of in situ depot-forming MNs using poloxamer gels. Transition is loaded into the MN pores, and in situ gels are developed at body temperature. Reproduced with permission from [64] under CCBY.

The fabrication of hydrogel-forming microneedles (HFMNs) is a detailed process involving several steps: mold development, hydrogel formulation, casting, and post-fabrication treatments. First, an MN-shaped mold is precisely created using advanced photolithography or micro-molding techniques. Next, biocompatible polymeric materials are carefully mixed with a suitable crosslinking agent to form a customized hydrogel. This hydrogel is then poured or injected into the mold cavities, with vacuum or centrifugation employed to eliminate any air bubbles. Afterward, the hydrogel is crosslinked, either chemically or physically, to ensure uniformity and structural integrity. Finally, post-fabrication treatments, such as dehydration, sterilization, and surface modifications, are applied to enhance drug loading, release properties, and mechanical strength [62,63]. Figure 5 illustrates a generalized technique for fabricating microneedles (MNs) using a poloxamer solution through a cold method. Researchers developed a sustained dermal drug delivery system utilizing thermoresponsive poloxamers, which formed depots within the skin micropores after MN application. The optimized MN arrays demonstrated good mechanical strength and sustained drug release for 96 h. This sustained release was further confirmed by confocal microscopy, which revealed a higher fluorescence intensity of fluorescein sodium within the skin tissue [64].

#### 4.2.1. Casting

Casting is a widely used method for developing hydrogel-forming microneedles (HFMNs). The process begins with creating a master mold in the desired MN shape using photolithography or microfabrication techniques. A hydrogel solution, containing water-soluble monomers, catalysts, and active ingredients, is then poured into this mold. Solidification occurs through crosslinking, typically achieved by UV exposure, heat, or a chemical agent. Once solidified, the MNs are extracted from the mold. The casting technique allows for the production of MNs with intricate shapes and sizes that can be tailored for specific applications. It also facilitates the incorporation of bioactive agents, such as drugs, peptides, or growth factors, into the hydrogel, enabling controlled release upon skin insertion. This method is favored for its simplicity, cost-effectiveness, and scalability, making it suitable for a variety of biomedical applications [65]. However, casting presents several challenges, including limited control over microneedle geometry, the risk of air bubble formation, and difficulties in achieving uniform consistency [63].

#### 4.2.2. Electrospinning

Electrospinning employs a high-voltage electric field to draw a biodegradable polymer solution, such as poly(vinyl) alcohol, into a thin jet. As the jet moves toward a collector, the solvent evaporates, causing the polymer to solidify into a fibrous mat. Hydrogel-forming microneedles (HFMNs) are subsequently created by coating these fibers with poly(vinyl) pyrrolidone or hyaluronic acid (HA). By adjusting parameters such as voltage, flow rate, and the distance between the needle and collector, microneedles of various shapes and sizes can be produced [50,66]. MNs produced through electrospinning have a high aspect ratio, meaning their length is significantly greater than their width, which enhances skin penetration efficiency. Additionally, the electrospun polymer allows for controlled release of drugs or active agents, as manufacturers can fine-tune the diameter, length, and porous structure of the MNs by adjusting the electrospinning parameters. However, the electrospinning process is relatively complex and requires specialized equipment [67].

#### 4.2.3. Micro-Molding Method

Micro-molding is a widely used method for preparing hydrogel-forming microneedles (HFMNs). Typically, polydimethylsiloxane (PDMS) casts are created by casting around a solid master template and curing at 70 °C for two hours. This process produces a negative micro-mold that can be reused to quickly fabricate multiple arrays of HFMNs, making it ideal for optimizing various parameters. In drug delivery applications, a two-step fabrication process is often employed, concentrating active ingredients at the tips of the HFMNs to enhance release efficiency [68]. Donnelly et al. employed laser machining controlled by a galvanometer and guided by pre-designed computer-aided designs to produce micro-molds. Their approach demonstrated the feasibility of producing and reusing these micro-molds for HFMN manufacturing, facilitating the development of specialized molds. In another study, Lutton et al. utilized injection molding to fabricate micro-molds. In their method, metal microneedle master templates were placed in an injection molding machine, where pre-mixed silicone elastomer was injected into the molds and subsequently cured to form the micro-molds [69].

Both casting and micro-molding techniques play crucial roles in the development of microneedles, each offering unique benefits that cater to different aspects of drug delivery technology. Table 2 summarizes the differences between these two techniques.

#### 4.2.4. Solution-Cast Micro-Molding in the Fabrication of MNs

Solution-casting micro-molding is a notable method for fabricating hydrogel-forming microneedles (HFMNs), combining the advantages of both solution-casting and micro-molding techniques. This method is particularly effective for developing microneedles that can deliver drugs dermally while maintaining the properties of hydrogels, such as biocompatibility and controlled release. Smith et al. developed microneedles using the solution-cast micro-molding technique (vac-and-fill) with polymers such as polyvinyl pyrrolidone (either 20% or 40%) and hydroxyethyl cellulose with glycerol. This simple and cost-effective micro-molding technique eliminates air entrapment and bubble formation, which can reduce the reproducibility of the method [70]. However, while solution-cast micro-molding offers numerous benefits, challenges include ensuring adequate mechanical strength for effective skin penetration and maintaining stability during storage and use.

#### 4.2.5. Additive Manufacturing in the Fabrication of MNs

Additive manufacturing, commonly known as 3D printing, has revolutionized the fabrication of microneedles (MNs), offering significant advantages over traditional manufacturing methods. This technology enables the development of customizable, complex MN arrays that can be tailored to specific applications in drug delivery. As technology advances, the integration of additive manufacturing techniques is likely to enhance the functionality and applicability of MNs in various biomedical fields. The inks used for hydrogel-forming MNs typically consist of polymers that can form hydrogels upon application, combined with other additives to enhance performance. Examples of these inks include dextran, hyaluronic acid, chitin, chitosan, methacrylate gelatin, Gantrez, alginate, poly(vinyl alcohol), poly(cyclopentyl methacrylate), polystyrene-block-poly(acrylic acid) (PSPAA), polylactic acid, poly(methyl methacrylate) (PMMA), polystyrene, and polycaprolactone [45]. Hydrogel-forming microneedles (MNs) are widely used due to their biocompatibility and swelling properties. The concentration of bio-ink used for these MNs is determined by several factors, including mechanical strength, swelling properties, biocompatibility, drug loading and release, crosslinking density, viscosity and printability, degradation rate, and penetration depth [45]. Baramee et al. investigated four different needle shapes: a pyramidal shape over a long cube, a cone mounted over a cylinder, a pyramidal shape, and a conical shape, using computer-aided design with varied compensated bases. The printed MN molds were developed using polylactic acid resin, and MN patches were further fabricated using hydroxypropyl methylcellulose and polyvinyl pyrrolidone. The results of tests for printing parameters (curing time, printing angle, and anti-aliasing), physical appearance, mechanical properties, and skin insertion ability revealed better physicochemical properties for the pyramidal shape over a long cube and the cone mounted over a cylinder compared to the pyramidal and conical shapes. This suggests that 3D-printed construct MNs could serve as an excellent alternative to conventional mold-based MNs [71]. In another study, Baramee et al. suggested that a 3D-printed MN construct could serve as an alternative to MNs prepared using conventional techniques by fabricating lidocaine HCl-loaded MNs for a faster onset of anesthetic action. Furthermore, this 3D construct was fortified with Oryza sativa L extract complex, demonstrating enhanced skin penetration via the transfollicular route, along with safety and efficacy for the management of keratinocytes [72].

### 4.3. Evaluation Methods for Hydrogel-Forming Microneedle Arrays

The materials, designs, and preparation methods are vital parameters that determine the properties of hydrogel-forming microneedles (HFMNs). Effective targeted delivery relies on optimal characteristics such as mechanical strength, biocompatibility, flexibility, hardness, degradability, skin permeation, and release kinetics of HFMNs. Additionally, factors like length and geometry, array configuration, coating options, and fabrication techniques—including micro-molding, 3D printing, and laser ablation—collectively contribute to the successful application of microneedles in drug delivery. Comprehensive evaluations necessary for microneedles encompass rheological characteristics of polymers, sol–gel transition properties, polymer–drug–polymer compatibility, morphology analysis, in vitro and ex vivo skin-swelling indices, water content, mechanical strength, insertion force over the skin, depth of penetration, in vitro permeation studies, and both in vitro and in vivo drug release assessments, as well as histopathology studies.

Rheological analysis of polymeric materials is crucial in developing and optimizing hydrogel-forming microneedles (HFMNs) as it provides essential information about the flow and deformation characteristics of polymeric hydrogels. These properties are pivotal in determining the suitability of hydrogels for microneedle fabrication and functionality. HFMNs utilize hydrogels that swell in moisture, making them ideal for dermal drug delivery. Consequently, key parameters such as viscosity (measured using a rheometer), elasticity, gelation kinetics, and swelling behavior are typically analyzed prior to HFMN development. This analysis ensures that the microneedles are effective, reliable, and suitable for their intended applications. As advancements in hydrogel technology continue, rheological analysis remains an essential tool for guiding the development of innovative and high-performance HFMNs [64].

Studies on drug–polymer compatibility are crucial for the development of hydrogel-forming microneedles (HFMNs). By examining the interactions between pharmaceuticals and the polymeric matrix, researchers can ensure the stability, effectiveness, and reliability of microneedle systems. This research focuses on evaluating the compatibility between therapeutic agents and the polymeric matrix used in hydrogels to guarantee effective drug delivery and optimal performance of the microneedle systems. Maintaining the therapeutic efficacy of medications is essential, as drug stability within hydrogel matrices can be compromised by incompatibility. Incompatible drug–polymer interactions may lead to a gradual deterioration or loss of drug potency, affect the chemical structure, or induce degradation, ultimately impacting the drug’s effectiveness. Additionally, the compatibility of the drug and polymer significantly influences the controlled release of the therapeutic agent. Various spectroscopic methods, including Fourier-transform infrared spectroscopy (FTIR) and nuclear magnetic resonance spectroscopy (NMR), as well as chromatographic techniques such as advanced high-performance liquid chromatography (HPLC), are employed to assess drug–polymer compatibility [48,73,74].

The transition of a material from a liquid (sol) phase to a gel (solid) phase is referred to as the sol–gel transition. This fundamental phenomenon is observed in polymeric and colloidal systems and is crucial for various applications, including drug delivery, materials science, and biological processes [75]. The development of enhanced delivery systems and various applications relies heavily on the texture, mechanical strength, and release characteristics of hydrogel systems, all of which are influenced by the sol–gel transition properties. Hydrogel-forming microneedles (HFMNs) utilize hydrogels that undergo this transition to create a stable three-dimensional network, facilitating effective drug delivery. Therefore, evaluating sol–gel properties is of prime importance. Various methods have been reported for this evaluation, including rheological measurements, microscopy, mechanical testing, swelling assessments, degradation studies, and biocompatibility analyses [64,76].

**Figure 6:**
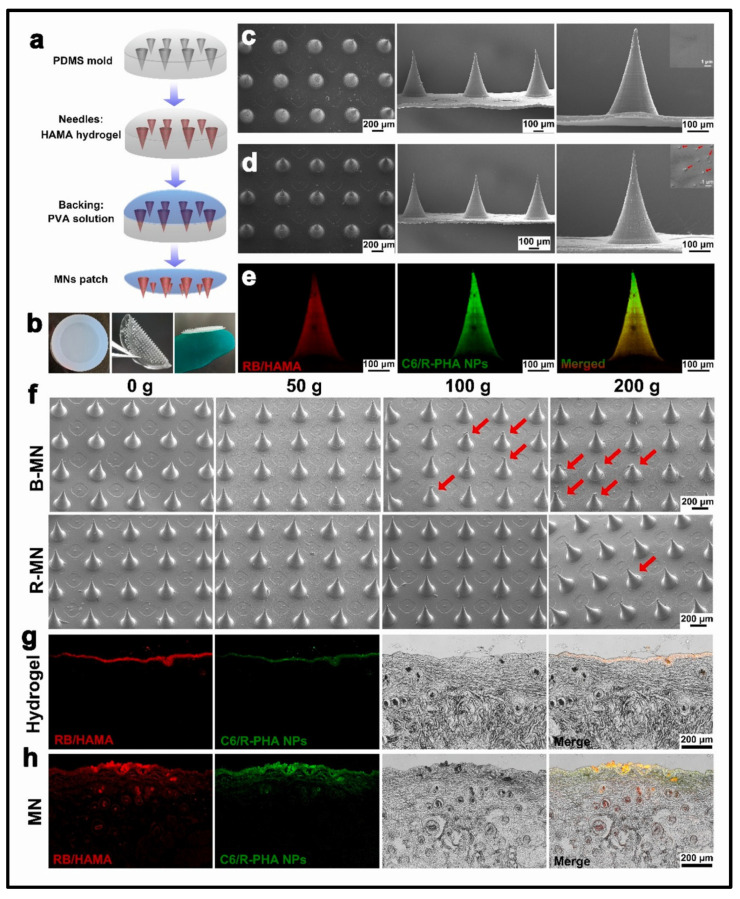
Illustrations of the fabrication process of HAMA-MNs (**a**) in PDMS mold (**b**). Scanning electron microscopy images of the blank HAMA hydrogel-forming microneedle (B-MN) patch (**c**), ritlecitinib–polyhydroxyalkanoate (R-PHA) NP-loaded HAMA-HFMN (R-MN) patch (**d**). Fluorescence microscopy images of the R-PHA NP-loaded HAMA-HFMNs (containing RB-labeled HAMA and coumarin 6-labeled R-PHA NPs) (**e**). Morphological changes in B-MN (blank HAMA-HFMN group) and R-MN (R-PHA NP-loaded HAMA-HFMN groups) after loading different mass weights (0, 50, 100, and 200 g) for 5 min (**f**). Representative fluorescence images of skin sections 24 h after topical application of fluorescence HAMA hydrogel (**g**) and fluorescence MNs (**h**), containing rhodamine B-labeled HAMA and coumarin 6-labeled R-PHA NPs. Reproduced with permission from [79] under CCBY-4.0.

Morphological examination of hydrogel-forming microneedles (MNs) is essential for understanding their structural integrity, functionality, and performance in applications. These tiny needles, designed to penetrate the skin’s outer layer, rely on hydrogel materials for effective delivery. Analyzing the morphology of MNs aids in comprehending their architecture, enhancing functionality, and ensuring efficacy in drug delivery and diagnostics. This evaluation confirms that MNs maintain their shape and mechanical strength during and after insertion into the skin. It also ensures that the MNs possess consistent dimensions, which is crucial for reproducibility and effectiveness. Additionally, morphological analysis assesses swelling behavior. Various methods, such as field emission scanning electron microscopy (FESEM) and transmission electron microscopy (TEM), are utilized to analyze the structure of MNs [61,77]. Figure 6 illustrates a scanning electron microscopy (SEM) image of microneedles (MNs) fabricated using polydimethylsiloxane (PDMS) miniature round molds. Each mold has a diameter of 17.5 mm, with a spacing of 700 µm between the centers of adjacent needle tips. The needle tips feature a bottom diameter of 270 µm and a height of 500 µm. The hydrogel-forming microneedles (HFMNs) are fortified with vascular endothelial growth factor and ritlecitinib to promote the proliferation and development of hair follicle cells, thereby enhancing angiogenesis [78].

**Figure 7:**
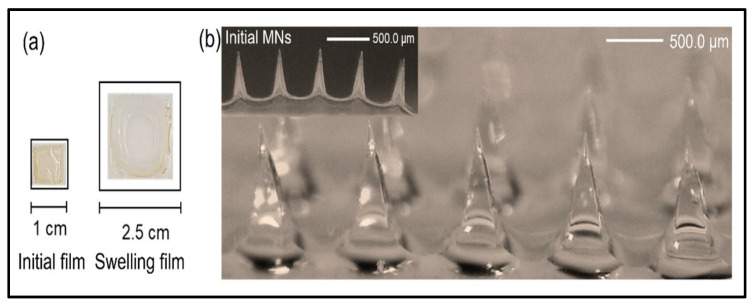
Swelling degree illustration for HFMNs in a comparative evaluation from the top view (**a**) and front view (**b**). Reproduced from [81] under CCBY-4.0.

One of the essential aspects of hydrogel-forming microneedle (HFMN) performance is the interaction with the skin, particularly regarding swelling behavior. Swelling studies are crucial for understanding how hydrogel MNs absorb interstitial fluid, expand, and release drugs [80]. Swelling behavior directly affects the release kinetics of therapeutic agents, and the extent of swelling influences the penetrability of microneedles (MNs) into the skin. To evaluate the swelling efficacy of the hydrogel-forming microneedle (HFMN) array, it is placed in contact with water and assessed at specific time intervals. Another method involves an ex vivo study using porcine skin, where the subcutaneous layer is removed. The HFMN patches are punctured into the isolated skin and then removed at designated time intervals to measure base-width capacity. Additionally, swelling is further tested concerning the water insolubility of HFMNs by placing the MNs in contact with water, followed by drying at 90 °C to compare the initial and final weights [81] (Figure 7).

Furthermore, the water content of hydrogel-forming microneedles (HFMNs) is crucial for their development and application, particularly in drug delivery and diagnostic systems. Water content influences various properties of hydrogels, including swelling behavior, mechanical characteristics, drug release rates, and overall stability. Therefore, accurate measurement and control of water content are essential to ensure the optimal performance and safety of microneedles. Swelling behavior significantly affects the effectiveness of hydrogels within microneedles, as higher water content typically results in more pronounced swelling. Mechanical properties also impact the ease of insertion and stability of microneedles. Additionally, appropriate water content can influence penetration and insertion, drug delivery efficiency, shelf life, and stability. By effectively managing these aspects, researchers and manufacturers can enhance the functionality and reliability of microneedle systems, ensuring effective performance and improved outcomes in medicinal applications.

For hydrogel-forming microneedles (HFMNs), mechanical strength is a crucial factor that impacts skin insertion, drug distribution, and overall durability. The primary objective of the mechanical assessment is to evaluate the resilience of microneedles to forces that could cause deformation, fracture, or failure. Key parameters in assessing mechanical strength include hardness, tensile strength, and fracture resistance. The mechanical strength of HFMNs is influenced by several factors, such as the composition of the polymeric hydrogel, manufacturing processes, and environmental conditions. Hardness measures a material’s resistance to indentation and abrasion, ensuring structural integrity and wear resistance during insertion and drug delivery. In contrast, tensile strength assesses the durability and flexibility of the material, while fracture resistance indicates its ability to withstand crack propagation and breakage under stress. Hydrogel composition and crosslinking density are also significant, with higher polymer concentrations typically enhancing strength. Additionally, manufacturing processes, including molding precision and drying conditions, can affect mechanical properties. Environmental factors, such as moisture and temperature variations, further influence these properties. Adhering to testing standards and implementing rigorous quality control measures are essential for optimizing microneedle design and performance [81,82].

**Figure 8:**
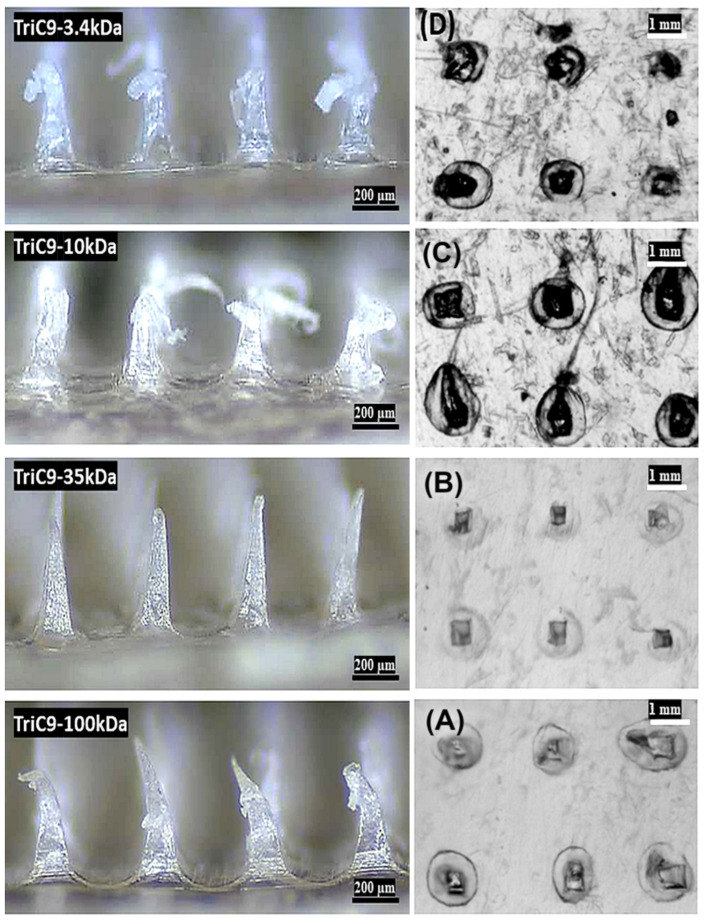
Insertion test in the Parafilm skin-simulant model. Percentage of holes created in the first two Parafilm layers by the MNs (**A**), height of MNs post-insertion to Parafilm layers (**B**), MN bending after insertion into the Parafilm layer (**C**), and the holes observed on the first layer by each MN patch (**D**). Reproduced with permission from [84] under CCBY-4.0.

Systems such as universal testing machines and custom force sensors are commonly employed to measure insertion force for microneedles (MNs). Various factors influence the required force, including MN design, geometry, needle tip shape, hydrogel properties, skin characteristics, hydration levels, and insertion speed. Evaluating comfort and user experience is critical, as lower insertion forces are generally associated with reduced discomfort for patients. Adherence to standardized testing protocols ensures consistency and reliability in measuring insertion force. By optimizing these parameters and following established testing guidelines, researchers and manufacturers can enhance MN performance, improve patient comfort, and ensure effective application in medical and diagnostic fields [83]. In a study conducted by Dawud et al. on hydrogel-forming microneedles (HFMNs) featuring programmed mesophase transitions for controlled drug delivery, the insertion force was tested on chicken skin at a vertical insertion rate of 1 mm/min until a desired normal force of 20 N was achieved. Following this, a dwell time of 30 s was maintained under the loaded state. The results indicated that an average insertion force of approximately 100 mN per needle was required when tested through a Parafilm skin-simulating model, as illustrated in Figure 8 [84].

**Figure 9:**
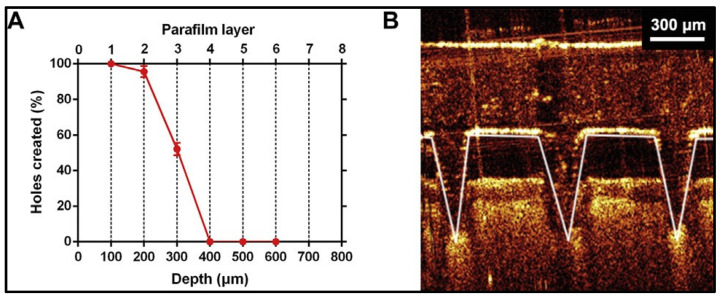
Illustration for insertion depth of MNs in (**A**) an artificial membrane and (**B**) rat skin using optical microscopy. Reproduced with permission from [78] under CCBY-NC-ND 4.0.

The depth of penetration of hydrogel-forming microneedles (HFMNs) into the skin is critical for effective drug delivery and diagnostic applications. Evaluating this depth is essential to determine the effectiveness and safety of microneedles in accessing the targeted layers of the skin [85]. Various measurement techniques are utilized to assess the depth of penetration of hydrogel-forming microneedles (HFMNs), including optical microscopy, histological analysis, advanced imaging techniques, and mechanical testing. Factors influencing penetration depth encompass microneedle design, hydrogel properties, skin characteristics, and insertion conditions. Generally, microneedles with longer lengths and larger diameters tend to achieve deeper penetration, while sharper tips enhance effectiveness [86]. For effective penetration, hydrogels with high swelling behavior and mechanical properties must be optimized. Skin characteristics, including thickness, elasticity, and the speed and pressure of injection, significantly influence penetration depth. Establishing evaluation protocols is crucial for obtaining reliable results in depth penetration studies. Standardized testing environments, which include consistent skin types, hydration levels, and insertion speeds, help ensure accurate and reproducible measurements. Safety considerations involve assessing potential tissue damage or irritation caused by microneedles during penetration. Histological analysis can identify adverse effects on skin tissues, while risk assessments evaluate the potential impacts of penetration depth on overall skin health and device performance [87]. Yu and colleagues developed layered dissolving microneedles (MNs) fortified with the immunosuppressant tacrolimus and diclofenac to alleviate skin and joint lesions in psoriatic arthritis. They tested the insertion depth on full-thickness rat skin using a force of 30 N for 5 min. Optical coherence tomography images, as shown in Figure 8 and Figure 9, indicated an insertion depth of approximately 300 µm through a 100 µm Parafilm layer covering the skin. Furthermore, the puncture hole ratio revealed a remarkable 100% pore creation, confirming that the thicknesses of the stratum corneum (SC), viable epidermis, and dermis are 10–20 µm, 50–100 µm, and 1–3 mm, respectively. This suggests that the fabricated HFMNs can effectively penetrate and deliver the loaded drug to deeper sites [78].

To understand how a drug is released from microneedles (MNs) over time, evaluating drug release profiles is essential. Typically, in vitro release experiments are conducted to assess the release profile, followed by fitting the data to various kinetic models, such as zero-order, first-order, Higuchi, and Korsmeyer–Peppas [43]. Furthermore, in vitro permeation studies are crucial for evaluating the effectiveness of hydrogel-forming microneedles (HFMNs) in delivering therapeutic agents across the skin. These studies provide insights into drug transport, formulation optimization, and safety compliance. The study involves several key components and procedures, including the selection of skin models, experimental setups, preparation of MNs, application to skin, and subsequent collection and analysis of data. While human skin is typically preferred due to its relevance to human applications, ethical concerns and availability may limit its use. Franz diffusion assembly is commonly employed to investigate drug diffusion across skin or membrane samples, utilizing either animal or synthetic membranes. The procedure includes loading the drug into the hydrogel matrix, curing the MNs, and ensuring they are adequately hydrated before application [88]. The application duration of hydrogel-forming microneedles (HFMNs) is optimized based on the type of microneedles used and the therapeutic requirements. Samples from the receptor compartment are collected at various intervals and analyzed to determine drug concentration. Understanding these methodologies and key parameters is essential for ensuring that MNs meet the required efficacy and safety standards. In vitro permeation studies are critical for evaluating the drug delivery capabilities of HFMNs and their effectiveness in clinical applications. Key parameters such as permeation rate, flux, lag time, and cumulative permeation provide valuable insights into the performance of the MNs. Factors influencing permeation include MN design, hydrogel properties, drug characteristics, and skin conditions. Generally, longer or sharper MNs achieve better permeation, while hydrogels with optimal properties facilitate effective drug delivery. Additionally, drug properties—such as solubility and molecular size—impact delivery efficacy, and skin characteristics—like thickness and hydration—can affect drug permeability. Pre-existing skin conditions or treatments may also influence permeation results, as can application parameters such as duration and pressure. Consistent application techniques are essential for obtaining reliable and reproducible results [61,88].

**Figure 10:**
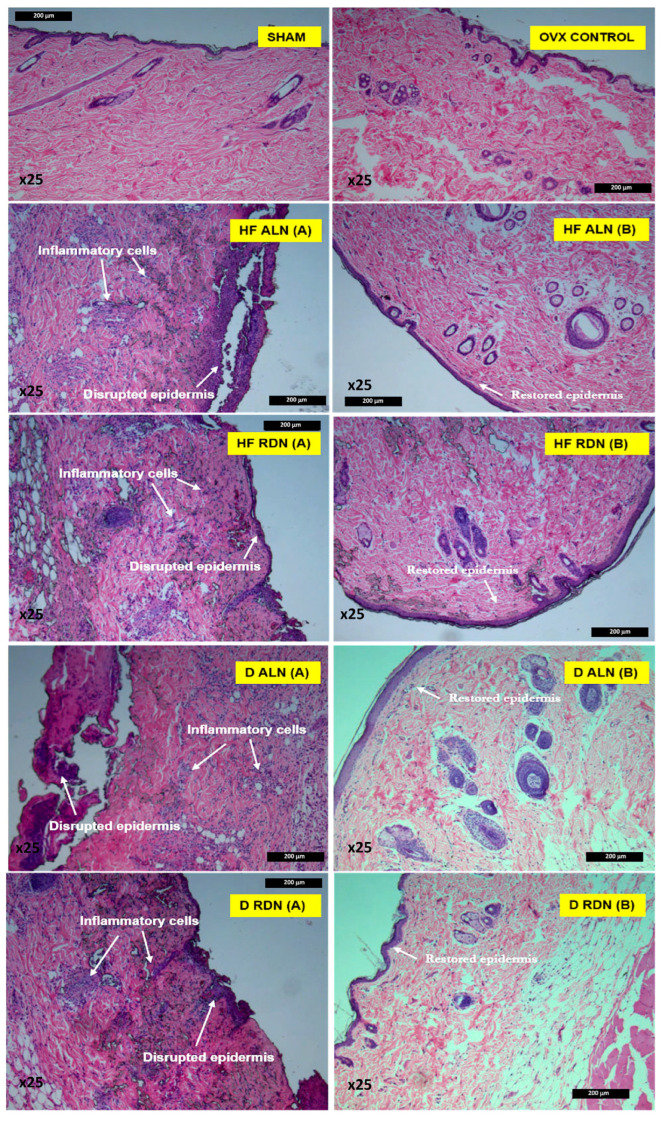
Annotated histological slides of SHAM and ovariectomy (OVX) control skin samples, as well as (**A**) regions of active inflammation for each MAP cohort, i.e., alendronic acid and risedronate sodium (RDN) [95] and (**B**) skin restoration images for post-application. Reproduced with permission from [96] under CCBY-4.0.

Histopathological studies are essential for assessing the safety and effectiveness of hydrogel-forming microneedles (HFMNs). These studies analyze tissue responses at a microscopic level to identify potential damage, irritation, or adverse effects. Understanding the results is crucial for ensuring that MNs are safe for use and perform as intended without causing significant harm to the skin. This detailed examination offers valuable insights into the biocompatibility and overall safety profile of MNs. Histopathology studies are vital for several reasons: they help elucidate the skin’s reaction to MNs, identify any inflammatory responses, and detect cellular damage or structural changes that may occur following MN application. Furthermore, these studies ensure biocompatibility by assessing whether the MNs and their components are compatible with skin tissues and do not induce harmful reactions [89]. Key parameters evaluated during histopathology studies include inflammation, cellular damage, structural changes, and the healing response. Several factors can influence the outcomes of these studies, such as microneedle design and properties, application parameters, the type and condition of the skin model used, and the duration of the study. Specifically, the design of the microneedles, along with how they are applied, significantly impacts the histopathological results, highlighting the importance of these variables in assessing the safety and efficacy of microneedle applications [90]. Ripolin et al. developed hydrogel-forming microneedles (HFMNs) fortified with alendronic acid and risedronate sodium to enhance the management of osteoporosis. In vivo testing on osteoporotic female Sprague Dawley rats demonstrated that the HFMNs significantly improved mean serum bone alkaline phosphatase levels, bone volume, and porosity compared to untreated bilateral ovariectomy controls. Furthermore, photomicrographs, as shown in Figure 10, revealed the active presence of inflammatory cells in the epidermis and dermis of animals treated with the microarray patch. Notably, the alendronic acid formulation exhibited more pronounced visible skin reactions and a greater presence of inflammatory cells in the dermis compared to the risedronate formulation [91].

Lastly, biocompatibility is a crucial evaluation parameter for hydrogel-forming microneedles (HFMNs), as it determines their safety and efficacy in medical applications. This assessment ensures that microneedles do not provoke harmful immune responses, cytotoxic effects, or other negative interactions with tissues. Evaluating biocompatibility is essential for confirming that HFMNs can be safely integrated into clinical settings without compromising patient health [92]. Biocompatibility is essential for several reasons, including safety, regulatory compliance, and effectiveness. To assess biocompatibility, various testing methods are employed, such as in vitro and in vivo tests, histopathological analyses, clinical studies, and long-term safety monitoring. These evaluations ensure that hydrogel-forming microneedles (HFMNs) interact safely with biological tissues and maintain their intended therapeutic effects without causing adverse reactions [92]. Key biocompatibility parameters for hydrogel-forming microneedles (HFMNs) include cytotoxicity, skin irritation, sensitization, inflammatory response, and the healing and repair processes. Several factors influence biocompatibility, such as material composition, microneedle design, application parameters, skin characteristics, and the testing methods employed. Material composition encompasses hydrogel properties, additives, and potential contaminants. Additionally, MN design, injection depth and pressure, duration of contact, and specific skin characteristics all play significant roles in determining the overall biocompatibility of the device [93]. Microneedle design, including factors such as size, shape, and surface properties, significantly impacts tissue penetration and the subsequent biological response. Application parameters, including injection depth, pressure, duration of contact, and specific skin characteristics, also play critical roles in determining biocompatibility. These elements collectively influence how the microneedles interact with the skin, affecting both safety and efficacy [94].

## 5. Application of Hydrogel-Forming Microneedles

In the realm of nanotechnology-enabled dermal drug delivery, hydrogel and in situ gel-forming microneedles (MNs) represent cutting-edge alternatives with significant potential for treating various skin-related conditions. These advanced MN systems enable controlled, precise, and efficient drug administration to specific skin layers, thereby enhancing therapeutic outcomes and reducing systemic side effects. HFMNs are designed to penetrate the stratum corneum and deliver therapeutic agents into the viable epidermis and dermis, offering promising applications for managing a range of skin-associated diseases. By targeting these layers directly, hydrogel and in situ gel-forming MNs improve the effectiveness of treatments while minimizing adverse systemic effects.

**Figure 11:**
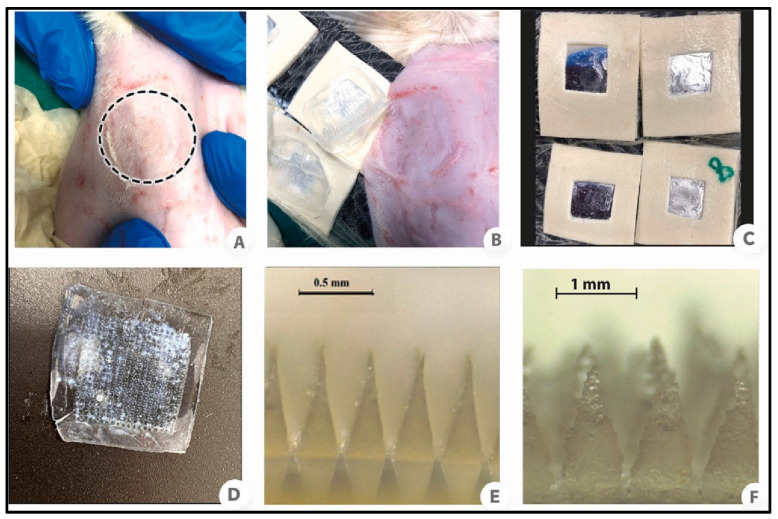
Macroscopic illustration captured after 24 h of HFMN applications and immediately upon HFMN removal, site of application of HFMNs (**A**), easy removal of swollen HFMNs in intact form (**B**), backing adhesive layer, where the reservoirs were inserted before their application (**C**), swollen HFMNs after removal from skin and backing layer (**D**), microscopic image of HFMNs prior to insertion into skin at scale bar of 0.5 mm (**E**), and microscopic images after insertion at scale bar of 1.0 mm (**F**). Reproduced with permission from [97] under CC BY 4.0.

Naser and co-workers developed poly(ethylene glycol)-based hydrogel-forming microneedles (HFMNs) utilizing a solid dispersion reservoir to deliver a micro-depot of atorvastatin (see Figure 11). The study revealed that approximately 2.05 mg of the drug was released from the dispersion over a 24 h period through an ex vivo diffusion compartment, maintaining therapeutic concentrations of over 20 ng/mL for up to 14 days. This indicates a tenfold increase in systemic exposure levels, which could enhance patient compliance significantly [97]. In another study, Courtenay et al. developed hydrogel-forming microneedles (HFMNs) for esketamine to address challenges associated with parental administration. Analysis of blood plasma samples revealed enhanced permeation of esketamine, demonstrating concentrations exceeding the required therapeutic range of 0.15 to 0.3 µg/mL over a 24 h period [98]. Turner et al. developed an array of hydrogel-forming microneedles (HFMNs) for the controlled delivery of amoxicillin and vancomycin. Their evaluation demonstrated potent antimicrobial activity against *Escherichia coli* and *Staphylococcus aureus*, highlighting HFMNs as a promising alternative to conventional formulations [99]. Jadach et al. fabricated an array of hydrogel-forming microneedles (HFMNs) coated with clotrimazole to treat fungal infections caused by skin mycosis. Their findings revealed that the application of clotrimazole effectively inhibited the growth of *Candida albicans*, as demonstrated by the suspension-plate technique. Additionally, the microneedles coated with clotrimazole dissolved in hydrogel exhibited superior antifungal action compared to conventional formulations [96]. Abraham et al. prepared hydrogel-forming microneedles (HFMNs) composed of rifampicin. Their findings revealed a dermal permeation profile that demonstrated approximately 500 μg of rifampicin penetration, along with a four-fold increase in rifampicin deposition (around 80 μg) within the skin layers. This study suggests that smart films can serve as innovative drug reservoirs for HFMNs, enhancing drug loading and diffusion characteristics through the hydrogel matrix [100].

**Figure 12:**
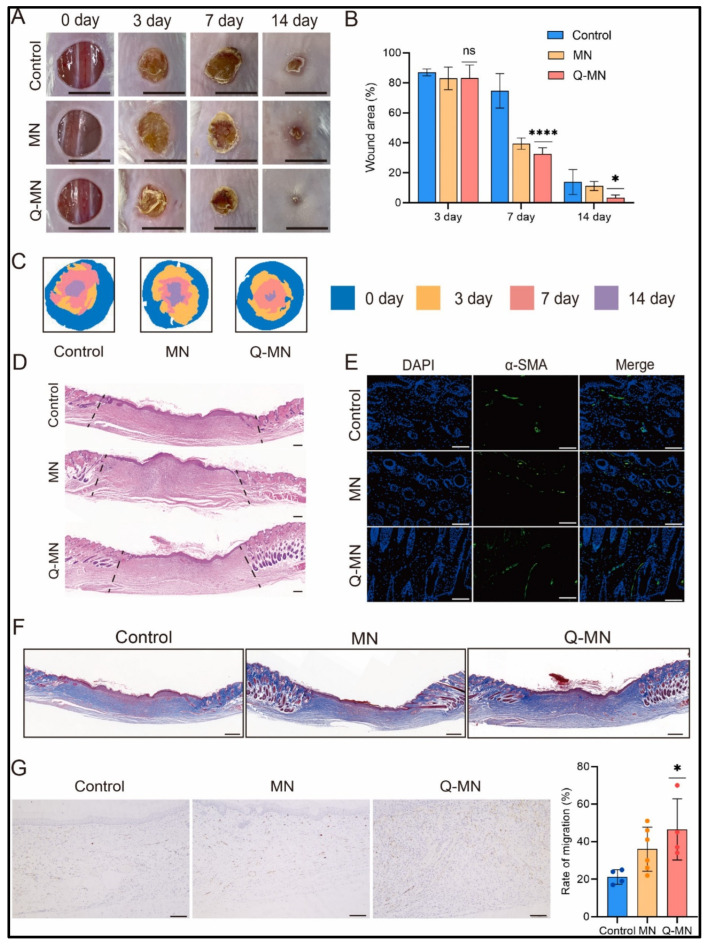
The illustration for wound healing progression activity. Microscopic image of wound in 3 groups (control, MN, and quercetin-loaded MN) at various time intervals in days (**A**), statistical representation of wound healing data (%) observed at various time intervals in days (**B**), wound healing progression representation (**C**), histo-morphological staining of wound tissue representation (**D**), immunofluorescence staining of wound tissue using α-SMA (**E**), Masson staining of wound tissue obtained on day 14 (**F**), neovascularization of wound at day 14 (scale bar of 30 μm) (**G**). Reproduced with permission from [104] under CC BY-NC-ND 4.0. * *p* < 0.05, **** p < 0.0001, ns, not significant.

Tekko et al. developed a hydrogel-forming microneedle (HFMN) array for the delivery of methotrexate, targeting arthritis treatment. The study found that the HFMNs exhibited mild swelling characteristics alongside acceptable mechanical strength, demonstrating effective insertion into excised newborn pig skin. The patch-like reservoir was loaded with a high dose of methotrexate (150.3 ± 5.3 µg/mg) without causing precipitation. An ex vivo study indicated that the integrated patch delivered methotrexate at a steady-state flow rate of 506.8 ± 136.9 µg/cm^2^/h [101]. In another study, Yadav et al. fabricated super swelling hydrogel-forming microneedles (HFMNs) for the delivery of ibuprofen sodium. In vitro diffusion experiments conducted over excised pig skin demonstrated the release of approximately 20% of the loaded ibuprofen sodium from the lyophilized wafer within 24 h. The findings suggest that enhancing drug transport through the skin can be achieved by increasing the solubility of ibuprofen sodium in the reservoir [102]. Mahfufah et al. developed a hydrogel-forming microneedle (HFMN) system for the delivery of albendazole to treat parasitic infections. Investigations into skin integrity showed no alterations due to the penetration of HFMNs into the skin. Furthermore, ex vivo permeation tests utilizing a polyethylene glycol (PEG) reservoir demonstrated a penetration of 4584.43 ± 26.61 µg/cm^2^ of albendazole into the skin [103]. Yi et al. developed HFMNs of GelMa encapsulated with quercetin to evaluate wound healing activity. It was demonstrated that quercetin HMNs aided in wound healing in both in vitro and in vivo settings, encouraging the production of collagen and neo-angiogenesis while decreasing oxidative stress. As a result, increased vascularization and collagen production were observed, suggesting that the given HFMN array can promote wound healing (Figure 12) [104]. Yao et al. developed an HFMN array patch loaded with stem cell-derived mitochondrial extracellular vesicles to improve wound healing. Metformin was used to enhance mitochondrial biogenesis. The findings reveal that the array patch enhances the healing process in in vivo examinations [105].

Interstitial fluid is used as an alternative to blood for quantifying the exogenous content administered; however, current strategies require sophisticated instruments that consume both time and resources for patients. Recent studies have demonstrated that MNs are a potential substitute for these methods. Li et al. developed surface-enhanced Raman spectroscopy MNs fortified with dye and mitoxantrone. In contrast, a gold–silver substrate with a hydrogel coating was used to compare painless, real-time analysis of drugs in dermal interstitial fluid after intravenous injection. The pharmacokinetic data suggested that mitoxantrone concentration was comparable in blood and dermal interstitial fluid, although the concentration of mitoxantrone in dermal interstitial fluid was 2–3 orders of magnitude lower than in blood, providing insightful information into the potential of dermal interstitial fluid as an alternative to blood for in vivo drug detection [106]. In another study, Razzaghi and co-workers developed MNs made of polyethylene glycol diacrylate as a device that acts as a biomarker in interstitial skin fluid within minutes of insertion, successfully managing metabolic disorders [107].

## 6. Clinical Updates and Challenges

Hydrogel-forming microneedles are emerging as a promising technology in the management of dermal disorders, particularly for drug delivery and patient monitoring. Recent advancements have highlighted their potential to enhance dermal drug delivery, minimize invasiveness, and improve patient compliance. Kasasbeh and co-workers conducted a clinical study involving repeated applications of an HFMN array made from a copolymer of vinyl ether and maleic acid crosslinked with polyethylene glycol. The findings revealed that, upon repeated application, no visible quantity of polymer was observed; however, some patients experienced mild erythema, which resolved within 7 days, confirming patient compliance with fewer or no side effects from the HFMNs [108]. In another study conducted by Donnelly and co-workers on HFMNs for enhanced dermal drug delivery, gamma-sterilized HFMNs were applied to the ventral forearm skin of six healthy human volunteers, aged between 23 and 31 years (three men and three women), who had no prior skin issues. Pain scores were recorded immediately after application of the patch, concluding that no adverse effects were observed after 24 h [23].

Hydrogel-forming microneedles (HFMNs) for dermal drug delivery face challenges related to manufacturing scalability, regulatory approval, patient acceptance, efficacy and dosage control, storage stability, integration with existing therapies, and cost-effectiveness. Advanced manufacturing methods, such as 3D printing and microfabrication, can enhance scalability and uniformity, while early collaboration with regulatory bodies can streamline the approval process. Patient education through clinical trials and public outreach can increase acceptance. Tailoring formulations and dosing strategies to specific populations can ensure consistent drug delivery and therapeutic effectiveness. Stability issues may be mitigated through research into more durable hydrogel compositions. Integrating HFMNs with existing treatments may require updates to clinical practices, supported by guidelines and evidence of efficacy. Cost challenges, particularly in resource-limited settings, could be addressed through cost-reduction strategies in manufacturing and partnerships with healthcare providers for initial subsidies. By focusing on these areas—research, regulatory alignment, patient education, and manufacturing innovations—HFMNs could become more widely adopted in dermal drug delivery.

As a potential advancement in dermal medication delivery, HFMNs have been utilized in both cosmetic applications and the management of chronic illnesses. However, to fully realize their potential, several obstacles must be addressed [21]. These challenges include regulatory compliance, skin variability, medication delivery efficiency, biocompatibility and safety, manufacturing and scalability, as well as environmental and sustainability concerns. Cost is a significant obstacle, and successful manufacturing and scalability depend on precise control over the hydrogel characteristics, size, and geometry of the microneedles. Long-term safety data are essential to prevent issues such as persistent inflammation, allergic reactions, or skin degeneration, making biocompatibility and safety critical considerations. Skin variability also affects performance, highlighting the need for regulated release and medication stability for effective drug administration. Moreover, regulatory approval processes can be complex, with local standards for quality, safety, and efficacy varying significantly [31].

## 7. Conclusions and Future Perspectives

Hydrogel-forming microneedles are gaining traction in the field of dermatology due to their innovative approach to managing dermal disorders. Hydrogel-forming microneedles enhance drug delivery by providing sustained release of therapeutic agents, making them a game changer in managing skin diseases. This innovative approach addresses the limitations of traditional dermal systems. The efficacy and safety of these microneedles have been improved through advanced fabrication techniques and the use of polymeric materials. Nevertheless, challenges remain that need to be addressed. More robust clinical data are required to validate their efficacy and safety across different patient groups.

Artificial intelligence, on the other hand, has shown tremendous potential in the design of precise drug-delivery systems, increasingly playing a pivotal role in the development of HFMNs by enhancing their design, fabrication, and applications. The integration of machine learning and artificial intelligence technologies facilitates more efficient and innovative approaches to microneedle development. AI can further assist in the fabrication of microneedles through predictive modeling, selecting optimal materials, and utilizing the best-fit techniques in HFMN development.

Hydrogel-forming microneedles (HFMNs) are advancing rapidly in material innovation and application. Biodegradable hydrogels are being developed to reduce environmental impact and enhance patient safety. Composite materials, including nanoparticles and fibers, are boosting mechanical strength and drug-loading capabilities. The application range for HFMNs is expanding to areas such as vaccine delivery, chronic disease management, cosmetic treatments, and integration with digital health. Smart delivery systems now incorporate sensors and microelectronics, enabling real-time monitoring and feedback. Telehealth applications allow for easy sample collection and remote monitoring. Additionally, new regulatory pathways are establishing clearer guidelines and standards for microneedle development and testing. These advances promise to improve the functionality, safety, and application scope of HFMNs in medicine and cosmetics.

Future research should focus on optimizing the design and materials of microneedles, exploring their applications in a broader range of skin conditions, and improving their biocompatibility and user comfort. Moreover, advancements in nanotechnology and material science could further revolutionize this field, leading to even more effective and patient-friendly drug delivery systems. As these technologies continue to progress, they have the potential to significantly enhance the quality of life of individuals with various skin disorders.

## Figures and Tables

**Table 1:** Various parameters that need to be considered while fabricating HFMNs. Reproduced with permission from [42] under CC BY 4.0.

Parameters	Size and Dimension
Length (*L*)	Vary between 500 and 800 μm depending on applicability
Diameter of needle base (*Db*)	Vary between 150 and 300 μm depending on applicability
Tip diameter (*Dt*)	<15 μm
Internal spacing (center to center) (IS_1_)	Vary between 400 and 450 μm depending on applicability
Interneedle spacing (edge to edge) (IS_2_)	Vary between 50 and 150 μm depending on applicability
Array size	Vary between 1 × 9 and 19 × 19 depending on applicability

**Table 2:** Summary of the differences between casting and micro-molding techniques in the development of MNs.

Microneedles Through Casting Technique	Microneedles Through Micro-Molding Technique
Pouring liquid into molds	Developing a negative mold
A broad range of materials is utilized	Specialized polymeric materials are utilized
Moderate precision is observed	High precision is observed
Relative process is faster for large scale-up	Slower but scalable in fabrication
Flexible in design	High accuracy in clinical use

## Data Availability

Data can be made available on request to corresponding authors.
